# Getting the upper hand in ALS

**DOI:** 10.1038/s41434-022-00314-9

**Published:** 2022-01-20

**Authors:** Clive N. Svendsen

**Affiliations:** Board of Governors Regenerative Medicine Institute Cedars-Sinai Medical Center, 8700 Beverly Blvd, AHSP 8th Floor, Los Angeles, CA 90048 USA

**Keywords:** Regeneration and repair in the nervous system, Neurological disorders

Ubiquitin is perhaps the most aptly described molecule—ubiquitous to tissues throughout the body. Multiple copies of ubiquitin (polyubiquitin) tag proteins for degradation and this plays an important role in various neurodegenerative diseases. This discovery earned a Nobel prize and has become a critical area of research and drug discovery. Genc et al. provide strong evidence that UCHL1—an enzyme crucial for regulating polyubiquinated proteins at multiple levels—may be able to reverse degeneration of upper motor neurons in amyotrophic lateral sclerosis (ALS), one of the most aggressive neurodegenerative diseases. ALS, defined by Charcot in 1869, has to involve the loss of both upper and lower motor neurons leading to paralysis of voluntary muscles over a period of 3–5 years. While ~10% of all ALS cases are associated with specific gene mutations involving C9orf72, TDP-43 and SOD1 and others, the majority are “sporadic” with no known genetic origin. ALS is also now considered a proteinopathy with TDP-43 as the central player.

Genc et al. first assessed UCHL1 knockout mice, which showed significant degeneration of the upper motor neurons, characterized by severe loss of spines along their apical dendrites [1]. Injections of AAV-UCHL1 in the corticospinal tract led to retrograde expression in the upper motor neurons and complete reversal of this morphological degenerative phenotype. While this is proof of concept of a technical approach to gene replacement, it would be expected as the function of the knocked out gene is simply being replaced. Of greater interest is when they applied the same over-expression of UCHL1 specifically in the upper motor neurons of mouse models for two distinct causes of ALS that involve mutations in TDP-43 and SOD1. Remarkably, they found very similar complete restoration of the upper motor neuron phenotype. UCHL1 expression also led to reduced levels of misfolded SOD1 and TDP-43 protein accumulation. This suggests a wider influence of UCHL1 activity on neuronal health across a range of mutations that cause ALS. With no known causal gene for sporadic ALS, genetic mouse models cannot be developed and tested with UCHL1. But, the inference is of course that increasing UCHL1 activity in sporadic ALS cases may also have beneficial effects.

While the ALS field has primarily concentrated on the lower motor neuron, of major interest here is the focus on the upper motor neuron. Signs of upper motor neuron involvement early in ALS comes from a number of pathological studies and some clinical studies showing that these neurons are hyperactive [2]. We have also been interested in this area and demonstrated that AAV-9 knockdown of mutant SOD1 only in the motor cortex was sufficient to delay disease onset and extend lifespan in a rat model of ALS [3]. This indicates that the cortex can be a target for therapeutic intervention, which was subsequently substantiated by our finding that delivery of neural progenitor cells engineered to produce glial cell line-derived neurotrophic factor (GDNF) into the SOD1 rat motor cortex can protect upper and lower motor neurons as well as delay disease progression and extend lifespan [4]. This provided the foundation for a current FDA-approved clinical trial to deliver GDNF-producing neural progenitor cells to the motor cortex of ALS patients.

Why such an interest in the upper motor neuron? It is perhaps one of the most unique cells in the animal kingdom. Located in layer 5 of the motor cortex, they project an axon over 1 meter down to the cervical and lumbar spinal cord in order to activate the lower motor neurons that connect to the muscle. During human evolution, these cells became further specialized to control fine and immediate thumb and finger movement. This necessitated a direct connection between these two motor neuron pools and allowed for the unique aspects of fine motor control [5]. In turn, this enabled humans to perfect tool production, writing and other unique attributes that led to modern day humans.

But this came at a cost. Rodents and other species maintained a mostly two step motor system, where the upper motor neuron connected first with a neuron in the brainstem that then activated the lower motor neuron. This dual functionality, where intermediate motor neurons in the brain stem permitted full motor control even in the absence of the upper motor neuron, was important for immediate “flight” responses without the complex cognitive process required to activate the upper motor neuron. Milliseconds of time difference in response could mean life or death to a small rodent and was probably a strong evolutionary advantage.

Further, the benefits of this direct upper to lower motor neuron in humans and the ability to perform complex movements may have had another cost. It is possible that the constant activation of lower motor neurons with no buffer or “automated” system in the brainstem could be stressful through excessive release of glutamate. In ALS this stress may be further enhanced through early degeneration of the apical dendrite of the upper motor neuron, and reduced inhibitory input leading to even more firing and perhaps increased stress on the lower motor neuron through this direct pathway. How this relates to TDP-43 biology remains to be established, but this is currently an area of great interest.

Could over-expression of UCHL1 in upper motor neurons affect this hypothesized process? Clearly the elegant work of Genc et al. suggests this is possible at the single motor neuron level. Retrograde tracing enabled the specific labeling of a few motor neurons that show very little pathology, while those that are not labeled are clearly degenerating in both selective UCHL1 knockouts and TDP-43 and SOD1 mouse models of ALS. But there were not enough affected motor neurons to expect an impact on the motor function of the animals. For this to occur, it would be necessary to affect more neurons by delivering more AAV-9-UCHL1 to the motor cortex—perhaps through cerebral spinal fluid administration or even convection-enhanced delivery directly to the motor cortex. However, even if delivered to the deep layer 5 motor neurons, there are other challenges. The AAV-9 UCHL1 construct used in this study was driven by the CMV promotor that would express in all cells. Would expression of UCHL1 in other types of neurons, astrocytes or microglial cells have any negative effects? If so, perhaps the AAV-9 would have to drive UCHL1 expression in just motor neurons requiring promotor-specific vectors that are often hard to develop.

Clearly the next stage involves a series of preclinical studies assessing dose ranging, efficacy and toxicity studies in rodent models and establishing a robust functional effect of UCHL1 delivery in the absence of any severe side-effects. This report provides a firm foundation for these types of studies and others diving deeper into the mechanism of UCHL1 protective effects on the upper motor neuron in human disease. However, given the unique features of this motor system in humans, the ultimate test may have to wait for well-controlled patient trials, as is often the case for complex neurological diseases. Based on the promising data shown in this report, direct AAV-based gene therapy to deliver UCHL1 to the motor cortex should be pursued as a therapeutic for ALS.
